# Evaluation of bacterial meningitis surveillance data of the northern region, Ghana, 2010-2015

**DOI:** 10.11604/pamj.2017.27.164.11036

**Published:** 2017-06-30

**Authors:** Basil Benduri Kaburi, Chrysantus Kubio, Ernest Kenu, Donne Kofi Ameme, Jacob Yakubu Mahama, Samuel Oko Sackey, Edwin Andrew Afari

**Affiliations:** 1 Field Epidemiology and Laboratory Training Programme, Department of Epidemiology and Disease Control, School of Public Health, College of Health Sciences, University of Ghana, Legon; 2 Ghana Health Service, West Gonja District Health Directorate, Damongo, Ghana; 3 Ghana Health Service, Northern Regional Health Directorate, Tamale, Ghana

**Keywords:** Bacterial meningitis, surveillance data, northern region of Ghana

## Abstract

**Introduction:**

Bacterial meningitis is a disease of major public health importance especially for countries such as Ghana; whose northern part lies within the meningitis belt. The Northern region of Ghana has been recording cases with outbreaks over the years. In order to generate evidence to improve surveillance, we described the epidemiology of bacterial meningitis using surveillance data of the northern region.

**Methods:**

Bacterial meningitis datasets from January 2010 to December 2015 for all the 26 districts of the Northern region were retrieved from line lists. Data were analyzed in terms of person, place, time, and identity of causative organisms using descriptive statistics. The results were presented as proportions, rates, tables and graphs.

**Results:**

A total of 1,176 cases were reported. Of these, 53.5% (629/1,176) were males. The proportion of cases aged 0 to 29 years was 77.4%. The Overall Case Fatality Rate (CFR) was 9.7% (114/1,176). About 65% of all cases were recorded from January to April. Only 23.7% (279/1,176) of cases were laboratory-confirmed. *Neisseria meningitides* and *Streptococcus pneumonia* accounted for 91.4% of confirmed cases. Over the period, the incidence reduced from 9.0/100,000 population to 3.8/100,000 population and CFR reduced from 16.6% to 5.7%.

**Conclusion:**

Most cases of bacterial meningitis were recorded in the dry season and in persons younger than 30 years. Less than a quarter of cases were laboratory confirmed, and no new bacteria species were identified. Both morbidity and mortality rates were on the decline. There is the need to consolidate these gains by intensifying meningitis surveillance and improving on the rate of laboratory case confirmation.

## Introduction

Meningitis is the acute inflammation of the meninges of the brain. The condition is termed Cerebrospinal Meningitis (CSM) if the meninges of both the brain and the spinal cord are involved. Meningitis is commonly caused by infectious agents such as: bacteria, fungi, viruses, parasites, and rickettsiae [1]. Non-infectious causes exist and include: drugs such as non-steroidal anti-inflammatory drugs (NSAIDs), neoplastic disorders, and sarcoidosis [1, 2]. Non-infectious meningitis can also result from post vaccination complications from vaccines against yellow fever, rabies, and rubella [2]. Inflammation of the meninges by non-pus producing bacteria and viruses results in aseptic meningitis; where the cerebrospinal fluid (CSF) is clear [2]. Meningitis is probably an age-long disease. Hippocrates (c.460 BC-370 BC) described a disease state very akin to meningitis. What is currently known as tuberculous meningitis was first described as "dropsy of the brain" by Robert Whytt (1714-1766) in a posthumous report [3]. The meningococcus as a causative agent for meningitis was identified by the Australian bacteriologist Anton Weichselbaum (1845-1920). In 1805, the first major outbreak of meningitis occurred in Geneva, Switzerland [4]. The 19^th^ century saw the disease spread through Europe, North America and Africa [5]. The first outbreak of meningitis to occur in Ghana (then Gold Coast) was at Cape Coast. Its source was traced to East African labourers brought in by the British colonial government to help fight the Ashantis. Since then, eight major outbreaks have occurred in: 1906, 1919/21, 1944/45, 1948/50, 1960/61, 1972/73, 1996/97, and 2009. By far, the 1996/97 outbreak remains the largest outbreak in Ghana [6]. The three Northern regions and the upper parts of Volta and Brong-Ahafo regions of Ghana fall within the African meningitis belt and are mostly affected. Sporadic focal outbreaks occur occasionally in other parts of the country; such as that of Asanti-Akim South in November 2008 [7].

Bacteria are the commonest cause of meningitis in immunocompetent persons. Nearly nine of every ten cases of post-neonatal meningitis are caused by three types of bacteria: *Streptococcus pneumoniae (S. pneumoniae), Neisseria meningitidis (N. meningitidis)*, and *Haemophilus influenza (N. meningitidis)* type b [8]. N. meningitidis and S. pneumoniae are those known to cause epidemic meningitis [5]. The mode of spread is by droplet infection from person to person. Consequently, overcrowding provides good conditions for the propagation of epidemics. People of low socioeconomic status usually live in overcrowded and poorly ventilated houses. Overcrowding is particularly worse in the harmattan season due to the cold nights. The day times of this season are characterized by dusty winds, high temperatures and low humidity. These conditions lead to breaks in nasopharyngeal mucosae, thereby increasing the risk of respiratory tract infections. Meningitis can complicate untreated or poorly treated upper respiratory tract infection [9, 10]. Other persons at risk of meningitis include the unvaccinated and the immune compromised such as those living with HIV/AIDS and diabetes mellitus [11]. Persons with acute bacterial meningitis typically present with a sudden onset of severe headache, fever, nausea, vomiting, nuchal rigidity and photophobia [12]. Globally, CFR of bacterial meningitis varies from 2% to 30% [13]. Within the African meningitis belt, the CFR in recent years has been relatively stable at about 10% [14]. In Ghana, institutional CFRs are between 36 and 50% [15]. About 10 to 20% of patients who recover from bacterial meningitis suffer debilitating neurological complications such as: epilepsy, mental retardation, and hearing impairment [8–14]. The adverse economic impact on already poor households, caused by either death or disability from meningitis is enormous [16]. Bacterial meningitis is preventable to a large extent. Effective vaccines are available against the most common causative agents [17]. These include *H. influenza* type b, *S.pneumonia*, and *N. meningitidis* serogroups: A, B, C, W135 and Y [17, 18]. There is also treatment available and depending on sensitivity results, antibiotic options for treatment include: benzyl penicillin, ampicillin, ceftriaxone, or chloramphenicol. These interventions not-withstanding, series of outbreaks continue to hit countries within the African meningitis belt. For example, the 2013 epidemic season recorded outbreaks that affected 18 of the 19 African countries with enhanced surveillance systems for meningitis. A total of 9, 249 suspected cases were recorded. This resulted in 857 deaths with a CFR of 9.3% by the 19^th^ epidemiological week [19]. Before 2010, Ghana suffered many meningococcal epidemics from strains A and C [20]. The first epidemic caused by W135 hit the Upper West Region in 2010 and followed by a second in the Upper East Region in 2012; with CFRs of 17.9% and 17.3% respectively [21, 22]. The Northern region has had its share of cases with focal epidemics within the Yendi municipality in 2014 that resulted in 83 confirmed cases and five deaths [23]. We therefore reviewed bacterial meningitis surveillance data of the region from 2010-2015 to describe the disease occurrence and the main causative agents in circulation in order to help improve bacterial meningitis surveillance in the region.

## Methods

**Figure 1:**
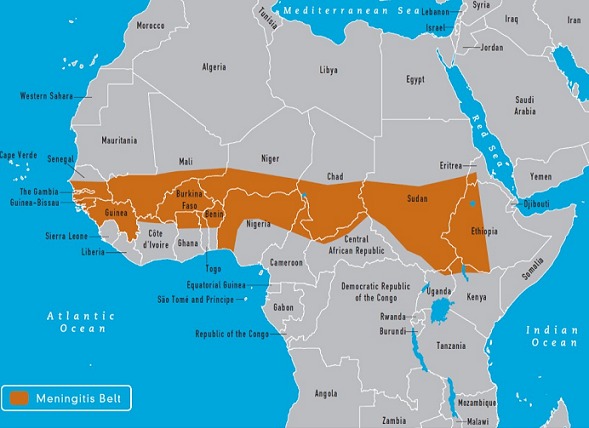
Map of Africa showing Northern Ghana as a part of the meningitis belt

**Figure 2:**
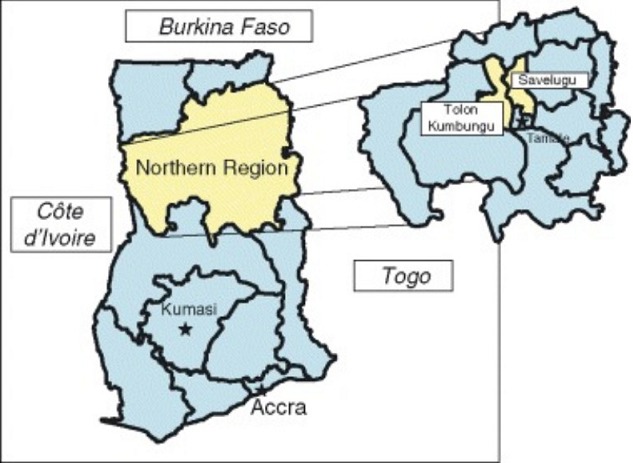
Map of Ghana showing the geographical boundaries of Northern Region

**Study area:** The Northern Region has a total land area of about 70,384 sq. km which is 29% of the land area of Ghana. It is located between latitude 8 30" and 10 30" N and lies completely in both the savannah and meningitis belts (Figure 1). It has Togo to the East and La Cote D´Ivoire to the West, as its international neighbours. It shares borders with Brong Ahafo and the Volta Regions to the south, and with Upper East and West Regions to the north (Figure 2). The region has 26 districts, 17 public hospitals, four (4) polyclinics, 24 clinics, and 107 health centres. Additionally, there are nine (9) private hospitals and 28 private clinics. The climate is tropical with temperatures ranging from as low as 9 degrees Celsius night temperature during the harmattan season to as high as 42 degrees Celsius during the hot dry season. The rains begin lightly in April and rise steadily to peak from August-September and gradually decline by the end of October. The dry harmattan winds engulf the whole region between December and February. These conditions enable cerebrospinal meningitis to thrive to almost endemic proportions.

**Study population:** The 2015 projected population of the region stands at 2,792,840 at a growth rate of 2.9 per annum. This is about 10.2% of the national population. The population density stands at 35 persons per sq.km. The population is characteristically distributed in small settlements with populations of 200 - 500 people. There are over 5,000 settlements in the region, out of which 54.4% have populations less than 200 people. The distances between settlements are long. This peculiar pattern of population distribution in the region has adverse implication for service delivery, For example, Sub-District Health Teams (SDHTs) going on out-reach services travel long distances only to reach a small proportion of their target population.

**Study design:** The study was a descriptive review of secondary data on all reported meningitis cases from January 2010 to December 2015. The study period was January and February 2016. We retrieved electronic versions of meningitis surveillance datasets captured as line lists in MS-Excel format. Key data elements extracted were age, sex, district, date of birth, date of onset of symptoms, date reported at the health facility, outcome of ailment, the type of bacterial meningitis and the serotype of *N. meningitides*. Using MS-Excel 2013, data was analysed in terms of person (age and sex), place (district), time (months and years), and types of causative organisms identified among confirmed cases. Trends were depicted by graphs and frequency distributions generated for key variables.

**Ethical considerations:** Authorities of Ghana Field Epidemiology and Laboratory Training Program (GFELTP) of the Department of Epidemiology and Disease Control, School of Public Health- University of Ghana and Ghana Health Service (GHS) granted permission for the access and use of the data for the evaluation. Permission was also officially sought from the Regional Director of Health Services for the use of the data. To ensure confidentiality, coded patient identification numbers were used in place of names and aggregate analyses done on data. Data held on computers were encrypted with a password which was made available only on a need to know basis.

## Results

**Figure 3:**
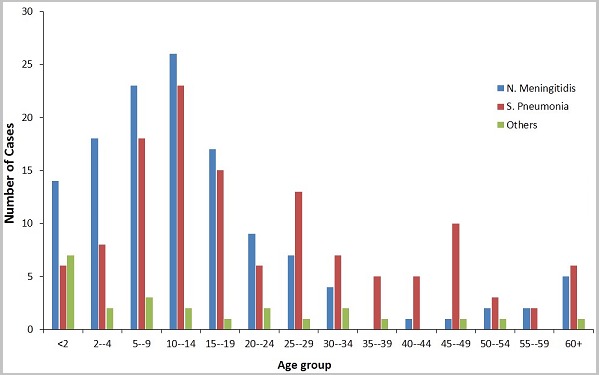
Distribution of causative organisms of bacterial meningitis by age, NR, 2010-2015

**Figure 4:**
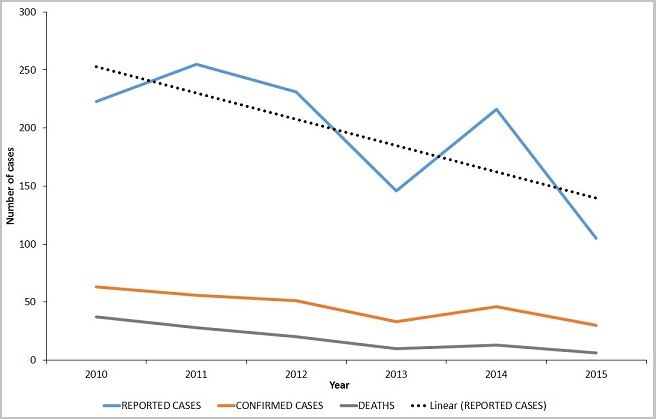
Trend of mortality and morbidity of bacterial meningitis cases, NR, 2010-2015

**Figure 5:**
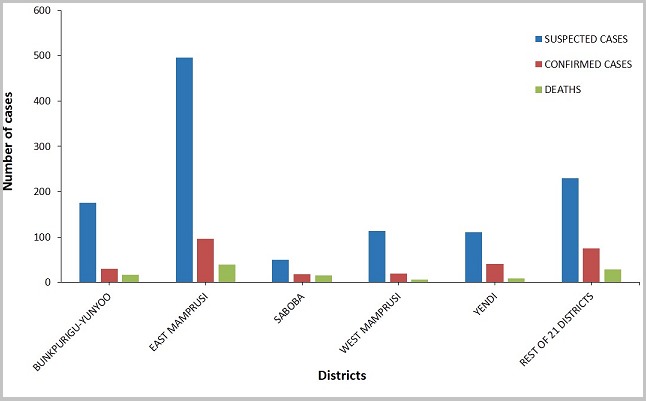
Distribution of bacterial meningitis cases by district, NR, 2010-2015

**Figure 6:**
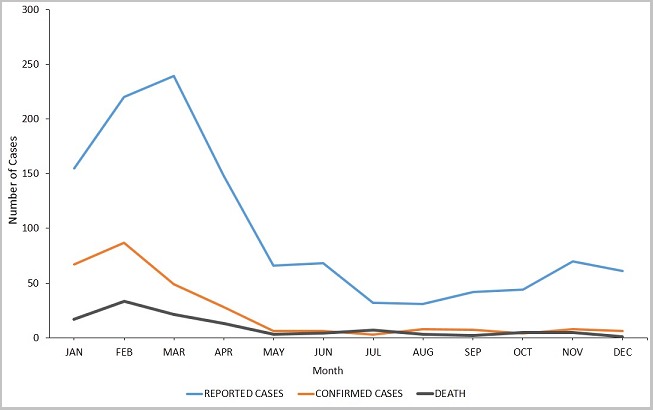
Cumulative seasonal trend of bacterial meningitis, NR, 2010-2015

A total of 1,176 suspected cases of meningitis were reported from all 26 districts for the 6-year period under review. The general trend of disease burden is presented in Table 1. Males constituted 53.5% (629/1,176) of the reported cases. Children under five years represented 27.4% (322/1,176), whilst those aged 60 and above represented 4.8% (57/1,176). The proportion of cases aged 0 to 29 years was 77.4% (910/1,176). Lumbar puncture was performed on 94.4% of all suspected cases reported within the entire period under review. The lumber puncture rate was 100% for three of the six years: 2012, 2013, and 2015. The rest were: 95.1% for 2010, 94.5% for 2011, and 81.0% for 2014. For the period under review, only 23.7% (279/1,176) of all the suspected cases were laboratory confirmed. Males constituted 51.6% (144/279) of the confirmed cases. By aetiology, 45.9% (128/279) were caused by *N. meningitidis*, 45.5% (127/279) by *S. pneumoniae*, and the remaining 8.6% (24/279) by a poorly identified group of bacteria labeled as "others" on the line lists. Of the 128 cases caused by *N. meningitidis*, only 36% (46) had further details on serotypes; indicating 44 for W135 and two for Nm C. No case caused by *H. influenza* was identified for the entire period. A total of 114 deaths were recorded; resulting in a CFR of 9.7%. The sex distribution of deaths was the same as the sex distribution of cases; 53.5% (61) of the deaths being males. Whereas *N. meningitidis* was the predominant cause of disease among all age groups from birth to 24 years, *S. pneumoniae* was dominant in all older cases as depicted in Figure 3. In the 2014 meningitis season, focal outbreaks of meningitis occurred in the Yendi municipality in which 83 confirmed cases and five deaths were recorded. Over the review period, there was a general downward trend of cases (Figure 4). Only five of the twenty-six districts (the pentad) accounted for 80.4% of reported cases, 73.1% of confirmed cases, and 75.4% of deaths (Figure 5). Four of these districts (East Mamprusi, Bunkpurigu-Yunyoo, Yendi, and Saboba all belong to the eastern corridor of northern region. East Mamprusi alone recorded 42.2% of all cases in the region. West Mamprusi district is an immediate neighbour of East Mamprusi. It ranks third in disease burden (9.6%), and completes the pentad. Most of the cases were recorded from January to April; about 65% (762) of the suspected cases (Figure 6) and 83% (231) of laboratory confirmed cases. This period also recorded about 74% (84) deaths from bacterial meningitis.

**Table 1:** Estimated annual incidence and CFR of bacterial meningitis in NR, 2010-2015

Year	Regional Population	Number of Cases	Number of Deaths	Incidence/100,000 Population	+CFR (%)
**2010**	2,479,461	223	37	9.0	16.6
**2011**	2,539,951	255	28	10.0	11.0
**2012**	2,602,431	231	20	8.9	8.7
**2013**	2,664,585	146	10	5.5	6.8
**2014**	2,728,081	216	13	7.9	6.0
**2015**	2,792,840	105	6	3.8	5.7

+ CFR-case fatality rate

## Discussion

The burden of bacterial meningitis is disproportionately distributed by place, time, and age but nearly equally distributed by sex. Whereas LP rates are high, the rate of laboratory confirmation of cases is low. The findings as presented are largely in keeping with the general literature on the epidemiology of meningitis along the African meningitis belt. Our findings reaffirm that in Northern Region of Ghana, bacterial meningitis is still endemic, affects all communities, both sexes, and all age groups. The disease distribution within the region in terms of person, place, time, implicated causative agents, and disease outcomes offer some insights to the strengths and weaknesses of the public health strategies against the disease. Over the period under review, whereas the population of the region grew steadily, there was a general downward trend in the estimated incidence of bacterial meningitis. As expected, most cases (77.4%) were younger than 30 years, and only 4.8% were older than 60 years. Children aged below 2 years represented more than a quarter of the cases. This is reported severally and attributed largely to their immature immune system [24]. Exposure of babies to perineal bacteria at delivery is also said to contribute to the higher risk of bacterial meningitis in the early neonatal period.

Accordingly, antibiotic prophylaxis for pregnant women against group B streptococci has been instituted in some developed countries [24, 25]. The CFR declined steadily over the period from as high as 16.6% in 2010 to 5.7% in 2015. This represents a 3-fold decrease in the number of deaths resulting from bacterial meningitis. Whereas the CFR for the first 2 years of the period under review are consistent with the 10-20% quoted for developing countries, the CFR for the later four years falls within the 5-10% estimated for developed countries [17]. Thus, the CFR of meningitis for these years have reduced and are now comparable to those of developed countries. This improved trend in morbidity and mortality justifies the investments directed at the prevention of bacterial meningitis in the last decade. These investments included the introduction of new polysaccharide and conjugate vaccines [26]. The implementation of robust vaccination programmes along with high quality clinical care has contributed significantly to the reduced burden of the disease in the region; just as similar programmes did in the advanced world about 2 decades ago. The decline in the incidence and CFR can be partly attributed to the improving literacy rates, rising public awareness of the epidemiology of the disease, and access to hospital care. Improved living conditions especially in terms of better housing facilities that have accompanied the rapid urbanization of the region have also played a part. The proportion of females in the region is about 51%. However, our study showed a higher proportion of laboratory confirmed cases (51.6%) among males. The predominance of cases among males was reported in a similar study conducted by Opare et al in neighbouring Upper East Region of Ghana in 2015 [27]. In a 12-year review of acute bacterial meningitis in Baltimore, Hussein and Shafran [28] found a higher prevalence among males, but the difference was not statistically significant [29]. Similarly, in their study of epidemiology of bacterial meningitis in the HSE Mid-Western Area in 2006, Whyte et al. found a statistically insignificant higher prevalence among males (p>0.05) [30].

*N. meningitidis* has long maintained its reputation as the aetiological agent responsible for most epidemics. In our study, the prevalence of *S. pneumoniae* (45.5%) among our cases was nearly the same as *N. meningitidis*(45.9%). There was however a clear predilection of *N. meningitidis* (38.4%) for cases 24 years and younger compared with *S. pneumoniae* (27.2%). This finding is consistent with results of similar studies conducted outside the meningitis belt by Namani and colleagues on causative pathogens of bacterial meningitis in children and their susceptibility to antibiotics [30], and Kyaw and colleagues on the changing epidemiology of bacterial meningitis [31]. In all age groups after 24 years, *S. pneumoniae* was the predominant aetiological agent. This is expected because the conjugate vaccines introduced against *S. pneumoniae* (PCV) is expected to reduce the burden of childhood pneumococcal meningitis substantially [32, 33].

The geographical distribution of cases is skewed to the eastern corridor of the region. Eight (8) out of every 10 cases come from only five of the 26 districts. These are: East Mamprusi, Bunkpurigu-Yunyoo, West Mamprusi, Yendi, and Saboba districts in order of decreasing disease burden. Indeed, East Mamprusi alone shoulders 42.2% of the disease burden of bacterial meningitis of the region. West Mamprusi does not belong entirely to the Eastern Corridor but is an immediate neighbour of East Mamprusi. Together, these five districts account for 3 of every 4 deaths from bacterial meningitis in the region. This disproportionate distribution of cases calls for studies to assess the possibility of the existence of additional risk factors in these populations that could account for this observation. Two well-defined seasons are recognized in Northern Ghana. These are: the rainy season (May to October), and the dry/harmattan season (November to April). The hot, dry, windy, and dusty weather conditions during the day times of the dry season increase the risk of communicable respiratory diseases [34, 35]. If these respiratory infections are poorly managed, meningitis could result. The cold nights of the harmattan season cause people to shut their windows overnight to keep warm. This results in poor ventilation. In an overcrowded room, these conditions facilitate the spread of infections from person to person. As anticipated, most cases occurred in the dry/harmattan season, with the peak incidence in March. About 66.2% of cases were recorded in these months (November to April) over the entire period under review. The region as a whole has not experienced an epidemic of bacterial meningitis during the period under review. However, focal epidemics were recorded in Yendi municipality in 2014. They were however well contained with reactive vaccinations and an intensification of public sensitization on the clinical presentation, risk factors pertinent to acquiring an infection, and preventive measures against disease acquisition and spread.

The aspect of laboratory investigation of specimens in the surveillance data analysed posed an important limitation not only for our study but for the public health surveillance system in the region. Despite an impressive 94.4% cumulative LP rate, aetiological agents were identified in less than a quarter (23.7%) of cases. Of the 128 specimens in which *N. meningitidis* was identified, only 46 had further details on the serotypes. Data on laboratory investigation was not available for 8.2% (96) of cases. Additionally, no organisms were said to be found in as many as 68.1% (801) specimens. The situations where no organisms were found could be due to the common practice of antibiotic abuse before presentation to hospital, or antibiotic administration in hospitals before specimens collection. It could also be that, collected specimens were not appropriately stored and/or transported prior to investigation. The aetiological agents for meningitis are fastidious organisms and would die easily under inappropriate storage conditions. These would then result in negative growths on cultures. This finding reveals a weakness in the adequacy of laboratory services in the region. This weakness may border on inadequacy of: laboratory infrastructure and logistics, personnel capacities and numbers, and the collaboration between level 1 and 2 laboratories on one hand, and the Zonal Public Health Reference Laboratory on the other. The confirmation of the specific causative agents among cases is central to effective clinical management and the implementation of preventive measures. Despite the availability of polyvalent vaccines, mutations and re-emergence of some aetiological agents can take the public health community by surprise. The enhanced meningitis surveillance remains an indispensable tool for early case detection, prompt confirmation of aetiological agents, and the identification of important epidemiological trends associated with the disease. Ultimately, the foregoing would collectively inform appropriate public health interventions.

**Limitation of the study:** The data described is based on reported cases which may not be accurately representative of numbers and distribution of bacterial meningitis cases in the Northern region.

## Conclusion

The highest proportion of reported meningitis cases was in the dry season, in patients younger than 30 years of age with a slightly higher prevalence in males. There is a disproportionate burden of the disease on the eastern corridor of the region. Both the incidence and CFR of bacterial meningitis in Northern Region have reduced over the study period. This suggests an improvement in epidemiological surveillance and case management. The laboratory surveillance data have been scanty and poor in quality. The findings support the need for more surveillance and better diagnostic services to further reduce significantly, the morbidity and mortality associated with bacterial meningitis.

### What is known about this topic

- The northern part of Ghana falls within the meningitis belt, therefore meningitis disease is a major issue of public health concern there;
- There have been outbreaks of meningitis in the northern region over the years.

### What this study adds

- There has been a reduction in incidence and case fatality rate of meningitis in the Northern part of Ghana over the past six years;
- Lapses have been found in the laboratory activities in the region and this need to be worked on in order to improve the surveillance system in the region.

## Competing interests

Authors declare no competing interest.
